# Individual differences in the language task-evoked and resting-state functional networks

**DOI:** 10.3389/fnhum.2023.1283069

**Published:** 2023-11-02

**Authors:** Xin Liu, Liu Yang

**Affiliations:** Air Force Medical Center, Air Force Medical University, Beijing, China

**Keywords:** functional magnetic resonance imaging, individual difference, language comprehension, functional network, resting-state fMRI

## Abstract

The resting state functional network is highly variable across individuals. However, inter-individual differences in functional networks evoked by language tasks and their comparison with resting state are still unclear. To address these two questions, we used T1 anatomical data and functional brain imaging data of resting state and a story comprehension task from the Human Connectome Project (HCP) to characterize functional network variability and investigate the uniqueness of the functional network in both task and resting states. We first demonstrated that intrinsic and task-induced functional networks exhibited remarkable differences across individuals, and language tasks can constrain inter-individual variability in the functional brain network. Furthermore, we found that the inter-individual variability of functional networks in two states was broadly consistent and spatially heterogeneous, with high-level association areas manifesting more significant variability than primary visual processing areas. Our results suggested that the functional network underlying language comprehension is unique at the individual level, and the inter-individual variability architecture of the functional network is broadly consistent in language task and resting state.

## 1. Introduction

The functional architecture of the human brain is characterized by notable inter-individual variability, which may underlie individual differences in cognition (Prat et al., 2012; Li et al., 2017; Jiang et al., 2020), personality (Feng et al., 2018, 2019), and emotion (Oudyk et al., 2019). A promising way to characterize the individuality of functional brain architecture is the analysis of functional networks, which estimate temporal synchronization of the blood oxygen level-dependent (BOLD) signals of separated brain areas (Ogawa et al., 1990; Finn et al., 2015; Jiang et al., 2020; Sui et al., 2020). Resting-state functional magnetic resonance imaging (rs-fMRI) is a common approach to non-invasively measure the spontaneous functional activities of the human brain (Biswal et al., 1995, 2010). Functional networks derived from the rs-FMRI data are posited to reflect intrinsic representations of functional systems commonly implicated in language processing (Koyama et al., 2010, 2011), cognition (Kong et al., 2019), and personality (Feng et al., 2018, 2019).

Notably, an increasing number of rs-fMRI studies have reported remarkable inter-individual variability in the resting-state functional network, and it indeed can serve as a fingerprint for individual identification (Finn et al., 2015; Waller et al., 2017; Tipnis et al., 2020). For example, Finn et al. (2015) applied the brain imaging data from the HCP to demonstrate that individual identification was successful across scan sessions and even between task and rest conditions, indicating that the functional organization within individual subjects is idiosyncratic and relatively robust to changes in brain state, and can be used to distinguish of individuals (Finn et al., 2015). In addition, Sbaihat et al. (2022) focused their analysis on the triple-network model (TNM), which is composed of the default mode network (DMN), the central executive network (CEN), and the salience network (SN), to investigate the intersession stability of the spontaneous functional network measures. They found strong stability for the regional homogeneity and the amplitude of low-frequency fluctuation in all three networks (Sbaihat et al., 2022). These findings indicated that spontaneous brain activity showed strong stability within an individual, and resting state functional parameters can be used as a promising way to predict individual traits. Moreover, a couple of previous studies focused their interests on functional network variability distribution, demonstrating that the functional variability is heterogeneously distributed across brain areas (Liao et al., 2017; Wang et al., 2021). For example, Liao et al. (2017) found that association networks, including fronto-parietal and attention systems, had higher modular variability, while primary sensorimotor and visual systems had lower variability (Liao et al., 2017).

Resting-state functional connectivity (RSFC) studies indicated that the functional systems observed during task performance are intrinsically represented in the brain by coherent low-frequency fluctuations in the BOLD signal within distinct functional networks (Biswal et al., 1995; Fox and Raichle, 2007). Nevertheless, the functional network variability related to a given task needs to be better investigated. Vanderwal et al. (2017) investigated the functional network variance architecture during a naturalistic movie-watching task. They indicated that the spatial distribution of inter-individual variability in the functional network during resting and movies followed the same pattern, that is, the lowest variability occurred in primary motor and sensory cortices, the greater variability occurred in the hetero-modal cortex, including the prefrontal and temporal cortices (Vanderwal et al., 2017). However, although studies have compared functional network variability during resting and natural task states, until now, inter-individual differences in functional network evoked by more constrained language comprehension tasks are still unclear.

Language comprehension is organized in large-scale brain networks of functionally interacting brain areas (Friederici and Gierhan, 2013; Hagoort, 2019). Previous works demonstrated that the language network is composed of a set of regions in the frontal, temporal, and parietal brain regions in the left and right hemispheres (Chai et al., 2016; Roger et al., 2022). According to a recent study, these areas can be divided into separate sub-networks responsible for coding-decoding, control-executive, abstract-knowledge, and sensorimotor functions, respectively (Roger et al., 2022). However, how individual functional connectivity differences vary across these sub-networks or brain areas is still generally limited. In addition, Although Vanderwal et al. (2017) have demonstrated that the functional network evoked by naturalistic movie-watching task generally shared a similar individual variability hierarchy as the resting state, a direct comparison between the constrained language comprehension task and resting state is still needed to give further evidence on domain-general and language-specific features of functional network variability architecture.

To address this issue, we employed the Human Connectome Project - Young Adults (HCP-YA; Van Essen et al., 2013) to explore the individual uniqueness of task-evoked functional brain network and its comparison between language task and resting state. In the language task derived from the HCP-YA, participants were instructed to either listen to a brief story and then answer subsequent comprehension questions about the story topic (story condition) or listen to arithmetic operation problems and indicate the correct answer (math condition). The story condition was implemented to engage in the rapid integration of conceptual information; therefore, its functional network was expected to elicit a network of brain clusters, which were thought to be responsible for the storage and retrieval of conceptual knowledge that underlies word and sentence meaning. We constructed individual functional networks in the resting state and the story condition of the language task state. We aimed to investigate: (1) whether resting and task state functional connectivity patterns are unique enough to capture individual variability; (2) whether there are any state differences in individual variability; (3) which brain functional module makes a dominant contribution to individual uniqueness, and is there any state differences.

## 2. Methods

### 2.1. Participants

Analyses were performed on a subset of 100 unrelated participants in the HCP-YA repository (Van Essen et al., 2013)^1^ to remove the influence of family-genetic factors on individual differences in brain function connectomes. All the participants were within the age range of 22–37 years at the time of scanning, and 46 of 100 participants were males, with no previously documented history of psychiatric or neurological disorders known to influence brain function.

### 2.2. The HCP-YA dataset

The HCP-YA data consists of 3 T MRI data, including structural (T1w and T2w), resting state fMRI, task state fMRI, and diffusion MRI modalities. There are seven tasks included in HCP-YA: emotion, gambling, language, motor, working memory, relational, and social tasks, respectively. We focused analyses on resting state and language task fMRI data. In HCP data acquisition, Oblique axial acquisitions alternated between phase encoding in a right-to-left (RL) direction in one run and phase encoding in a left-to-right (LR) direction in the other run, resulting in two runs (LR and RL runs) in both resting and task state fMRI data.

The resting state fMRI data were acquired in separate sessions (rest1 and rest2), with LR and RL runs in each session. The participants were instructed to stay relaxed and keep their eyes fixed on a projected bright crosshair on a dark background. There were 1,200 frames obtained in each run.

The language task in the HCP-YA dataset was developed by Binder et al. (2011). The task consisted of LR and RL runs. Each interleaves four blocks of a story condition and four blocks of a math condition as a baseline. The story blocks presented participants with brief auditory fables (5–9 sentences) followed by a two-alternative forced-choice question about the topic of the fable. For example, after a story about an eagle that saves a man who had done him a favor, participants were asked the following question: “That was about revenge or reciprocity?” In the math blocks, participants were aurally presented with a series of arithmetic operations trials, which were designed to match the length of the story task blocks, and also completed with two alternative questions about the correct answer of the math operations. For example, “Four plus twelve minus two plus nine equals twenty-two or twenty-three?” In both blocks, participants pushed a button to select either the first or the second choice. There were 316 frames per run.

### 2.3. Image acquisition

In the HCP dataset, whole brain high-resolution (2.0 mm isotropic voxels) fMRI images were acquired using a customized Siemens Skyra 3-T scanner with a 32-channel head coil. A gradient echo EPI sequence was used with the following imaging parameters: TR = 720 ms, TE = 33.1 ms, flip angle = 52°, FOV = 208 × 180 mm, slice thickness = 2.0 mm, 72 slices, with a multi-band acceleration factor of 8.

Structural scans included T1w and T2w scans. Parameters of T1w structural scans were: TR = 2,400 ms, TE = 2.14 ms, flip angle = 8°, FOV = 224 × 224 mm, voxel size = 0.7 mm. T2w structural scans were acquired using TR = 3,200 ms, TE = 565 ms, FOV = 224 × 224 mm, voxel size = 0.7 mm, with a variable flip angle. The WU-Minn HCP manual and HCP scan protocols overview the MRI acquisition details.^2^

### 2.4. Preprocessing

Preprocessing of all task and resting-state functional scans were performed using the HCP minimal preprocessing pipeline, including artifact removal, motion correction, and registration to standard space (Glasser et al., 2013). Three additional approaches were used for further processing of the fMRI data. One involved nuisance regression; this step was carried out for the task fMRI only, 24-parameter motion regressors, average time-series from the cerebrospinal fluid (CSF), and the white matter were regressed. The second is global signal regression, involving the removal of the global signal from the time series of each voxel using linear regression, which was applied for both fMRI modalities. The last is bandpass filtering; we filtered the time series with a minimum frequency of 0.009 Hz, maximum frequency of 0.08 Hz for resting state, and 0.25 Hz for task state fMRI.

### 2.5. Network construction

**Figure 1:**
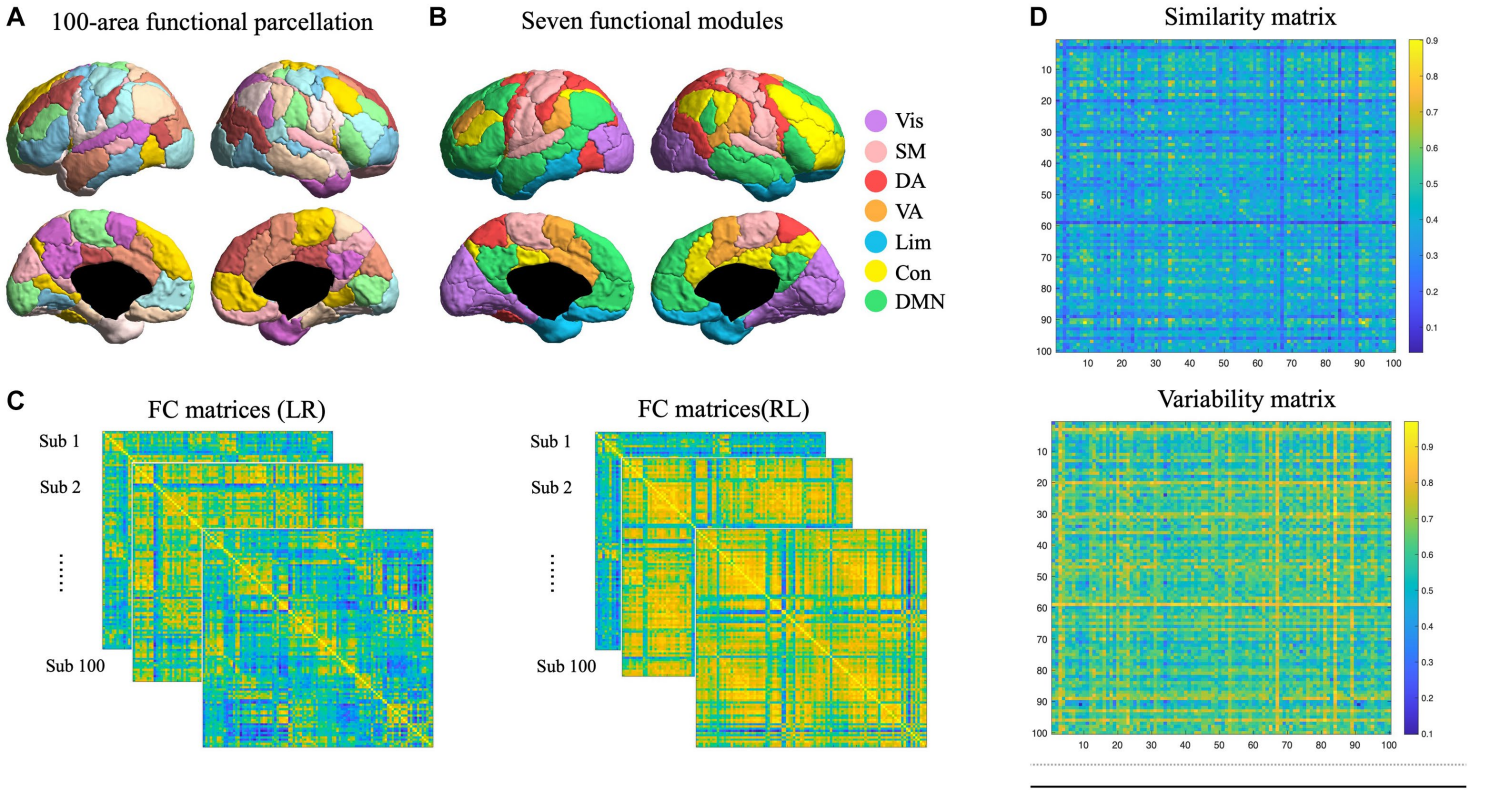
Functional network construction and variability quantification. **(A)** Functional parcellation of human cortex. A hundred cortical nodes were taken from Schaefer et al. (2018). **(B)** Nodes were divided into seven modules, including visual, somatomotor, dorsal attention, ventral attention, limbic, control, and default-mode network (DMN) in each hemisphere, which were marked in different colors. **(C)** Functional networks were constructed for each participant in LR and RL directions. **(D)** Individual similarity and variability matrices. The similarity matrix was constructed by the Pearson correlation between all edges in LR and RL connectomes, and the variability matrix was estimated by the inverted similarity matrix (1- similarity matrix).

Task and resting state networks were constructed for each participant. Nodes were defined by using a functional brain atlas developed by Schaefer et al. (2018), with different levels of granularity (100 brain nodes; Figure 1A). These nodes were further organized into seven modules. There were 17, 14, 12, 5, 13, and 24 nodes in visual, somatomotor, dorsal attention, ventral attention, limbic, control, and default-mode network (DMN) respectively in each hemisphere (Figure 1B). Edges were defined as Pearson’s correlation coefficients between each pair of regional time series, resulting in a 100*100 matrix of task and resting state, respectively, for each participant, respectively, (Figure 1C). In resting state, we extracted the time series corresponding to each brain node of the parcellation by averaging all the voxel-level time series belonging to each node. In the task state, we extracted time series corresponding to story conditions to ensure signal purity to language processing, as described in a previous study (Liu et al., 2017). The functional networks of rest 1 and rest 2 were averaged in resting state network construction. Functional networks were calculated for LR and RL runs, resulting in 4 networks for each individual (task vs. resting; LR vs. RL).

### 2.6. Functional network variability analysis

To quantify individual variability, first, we constructed a similarity matrix between LR and RL runs, and resting and task states, respectively, and then the inverted similarity was used to estimate network variability. This algorithm has been used in human (Gratton et al., 2018) and mouse (Bergmann et al., 2020) studies to estimate individual similarity and variability of functional networks. The similarity matrix was constructed by the Pearson correlation between all edges in LR and RL connectomes (Figure 1D). The variability matrix was estimated by the inverted similarity matrix (1- similarity matrix; Figure 1D).

Columns and rows in the variability matrix represent the LR and RL runs, respectively; values along the diagonal indicate the inverted correlation between the LR and RL connectomes of the same individual, which hence represent individual variability (intra-sub variability), the values remaining in the matrix represent the group variability (inter-sub variability), which indicate the inverted correlation between the LR of one individual and RL of other individuals. We conducted a paired sample t-test to examine the average inter- and intra-sub variability difference.

To test whether the individual variability differs between the seven modules, we constructed the similarity and variability network limited to each module. In the variability matrix of a module, values along the diagonal indicated the inverted correlation between edges of the LR and RL networks of the same individual, which hence represented intra-sub variability; the values remaining in the matrix represented the inter-sub variability, which indicated the inverted correlation between the edges of the LR of one individual and the edges of the RL of other individuals. Then, we compared the intra- and inter-sub variability across all modules by carrying out a one-way ANOVA, and all values were corrected for multiple comparisons using Bonferroni correction.

### 2.7. State comparison in functional network variability

To examine the difference between the resting and task states in functional network variability, we used a paired-sample t-test to compare the intra- and inter-sub variability values in resting and task states, respectively. Further, we carried out a 2 (resting/task) by 7 (visual/somatomotor/dorsal attention/ventral attention/limbic/control/DMN) two-factor ANOVA to investigate the state and module effect on intra- and inter-sub variability.

## 3. Results

### 3.1. Individual variation in resting state functional network

The variability matrix, calculated by the inverted similarity matrix between LR and RL functional networks, was used to quantify individual variation in the functional network by comparing intra- and inter-sub network variability. We conducted a paired sample t-test to examine the average inter- and intra-sub variability difference.

In the resting state functional network, inter-sub variability (mean *r* = 0.73) is significantly greater than intra-sub variability (mean *r* = 0.65; two-tailed paired Student’s *t*-test: T(99) = 9.00, *p* < 0.001). The significant difference between inter- and intra-sub network variabilities demonstrated that connectivity matrices from different individuals are more variable than connectivity matrices from the same individual in resting states, indicating substantial differences among participants and demonstrating that resting-state functional networks can capture individual variability.

To better characterize individual variation in all modules (visual, somatomotor, dorsal attention, ventral attention, limbic, control, and DMN), we sought to examine whether functional connectivity architecture within these modules differs in individual variabilities. Therefore, we defined each node in one of these seven modules. There were 17 nodes in the visual module, 14 in somatomotor, 15 in dorsal attention, 12 in ventral attention, 5 in limbic, 13 in control, and 24 in DMN, respectively, and we derived seven variability matrices accordingly. Then, we performed the variability analysis described above and compared variability across all modules by carrying out a one-way ANOVA. All values were corrected for multiple comparisons using Bonferroni correction.

**Figure 2:**
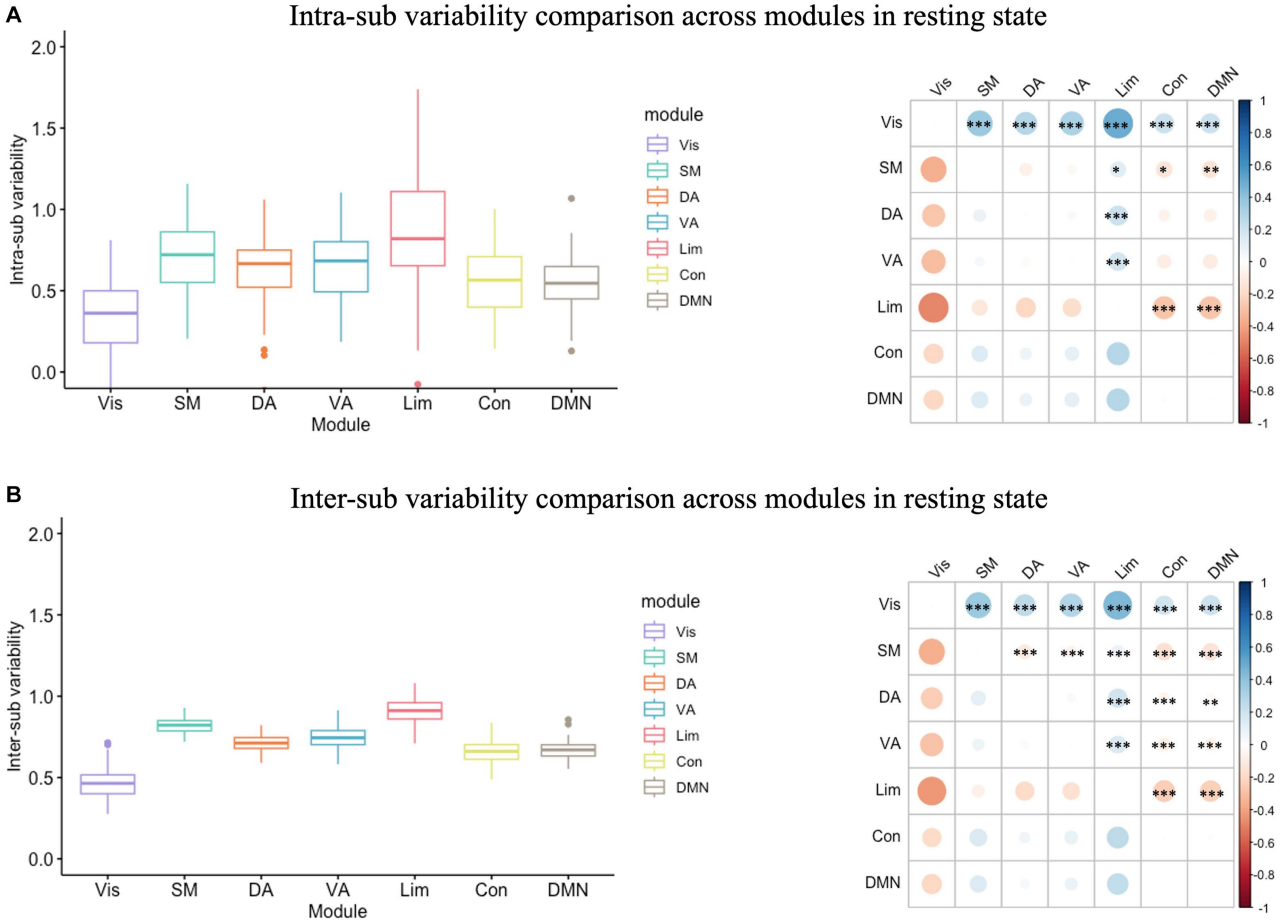
Intra- and inter-sub variability comparison across modules in resting state. **(A)** Intra-sub variability comparison. The lower line of the box indicated the first quartile of the inter-sub variability in each module; the upper line of the box indicated the third quartile of the inter-sub variability in each module; the middle line in the box indicated the median of the inter-sub variability in each module; the dots outside the box indicated the outliers of the inter-sub variability in each module; the line in the middle of the box indicated the range of the inter-sub variability in each module. The color bar indicated the difference between the intra-sub variability of the modules in the rows minus the intra-sub variability of the modules in the columns. The red dot on the right bubble matrix indicated that the intra-sub variability of the module on the row is greater than the corresponding module on the column; the blue dot on the right bubble matrix indicated that the intra-sub variability of the module on the row is less than the corresponding module on the column; the asterisks on the dot indicated the difference between the corresponding modules were significant. The limbic module showed the highest intra-sub variability, followed by the somatomotor, attention, and control modules, while the visual modules showed the most minor intra-sub variability. **(B)** Inter-sub variability comparison. Similar to the intra-sub variability comparison, the limbic module showed the highest inter-sub variability, followed by the somatomotor, attention, and control modules, while the visual modules showed the most minor inter-sub variability. Vis, visual module; SM, somatomotor module; DA, dorsal attention module; VA, ventral attention module; Lim, limbic module; Con, control module; DMN, default-mode network.

In the resting state functional network, the inter-sub variability was significantly greater than the intra-sub variability in each module (Supplementary Figure S1; Supplementary Table S1). In addition, there was a significant module effect in intra-individual variability (*F*(6) = 38.40, *p* < 0.001; Figure 2A) and in inter-sub variability (*F*(6) = 438.30, *p* < 0.001; Figure 2B). The following Tukey HSD test revealed that the limbic module showed the highest intra- and inter-sub variability, followed by the somatomotor, attention, control and DMN modules. In contrast, the visual modules showed the least intra- and inter-sub variability.

### 3.2. Individual variation in language state functional network

As for the language task state functional network, we also observed that the inter-sub variability (mean *r* = 0.61) is significantly greater than the intra-sub variability (mean *r* = 0.48; two-tailed paired Student’s *t*-test: T(99) = 12.64, *p* < 0.001).

**Figure 3:**
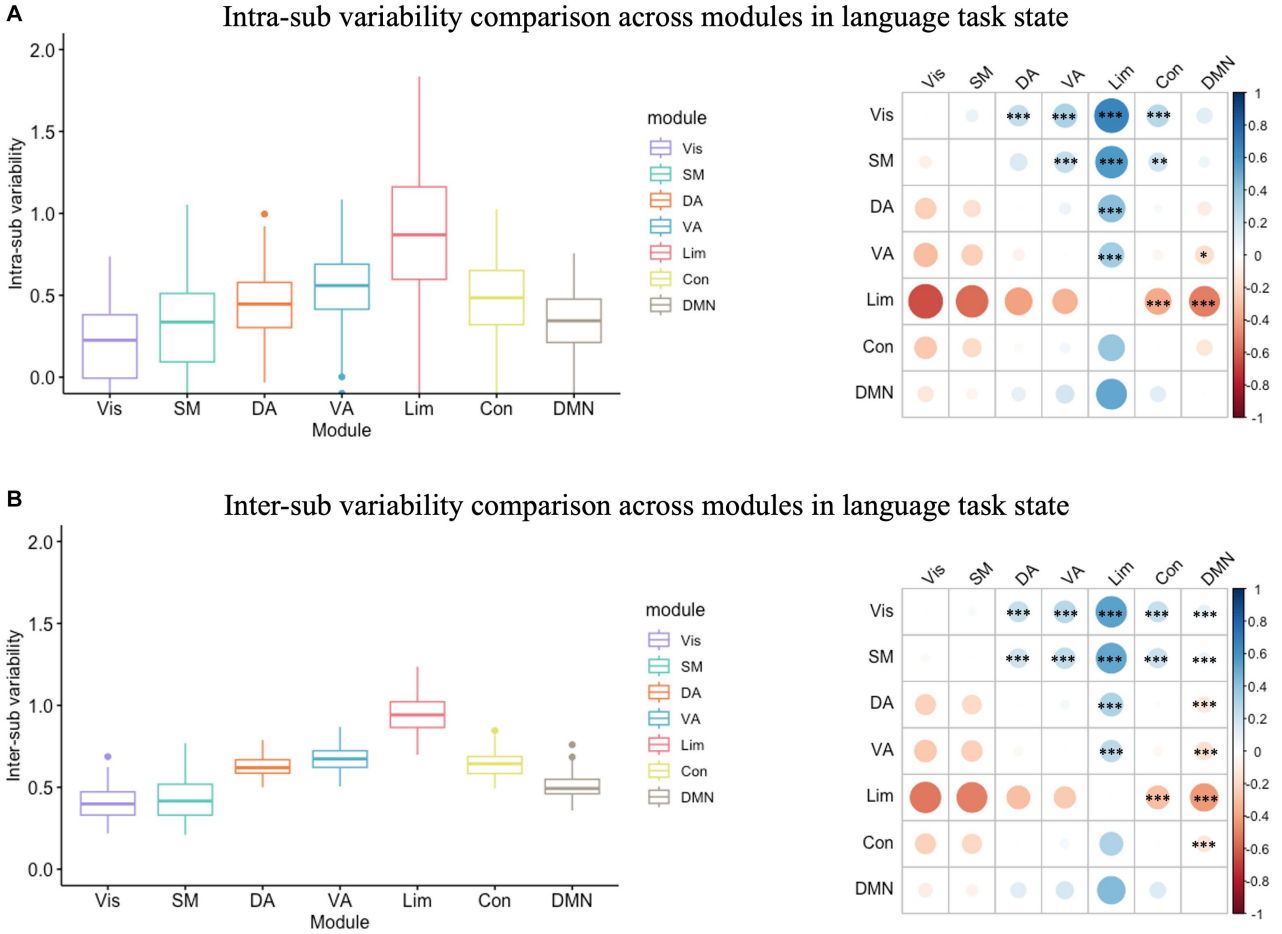
Intra- and inter-sub variability comparison across modules in the language task. **(A)** Intra-sub variability comparison. The lower line of the box indicated the first quartile of the inter-sub variability in each module; the upper line of the box indicated the third quartile of the inter-sub variability in each module; the middle line in the box indicated the median of the inter-sub variability in each module; the dots outside the box indicated the outliers of the inter-sub variability in each module; the line in the middle of the box indicated the range of the inter-sub variability in each module. The color bar indicated the difference between the intra-sub variability of the modules in the rows minus the intra-sub variability of the modules in the columns. The red dot on the right bubble matrix indicated that the intra-sub variability of the module on the row is greater than the corresponding module on the column; the blue dot on the right bubble matrix indicated that the intra-sub variability of the module on the row is less than the corresponding module on the column; the asterisks on the dot indicated the difference between the corresponding modules were significant. The limbic module showed the highest intra-sub variability, followed by the control, dorsal, and ventral attention modules, and then the DMN module, while the visual and somatomotor modules showed the most minor intra-sub variability. **(B)** Inter-sub variability comparison. Similar to the intra-sub variability comparison, the limbic module showed the highest intra-sub variability, followed by the control, dorsal, and ventral attention modules, and then the DMN module, while the visual and somatomotor modules showed the most minor inter-sub variability. Vis, visual module; SM, somatomotor module; DA, dorsal attention module; VA, ventral attention module; Lim, limbic module; Con, control module; DMN, default-mode network.

In the language task, the inter-sub variability was also significantly greater than the intra-sub variability in all modules (Supplementary Figure S2; Supplementary Table S1). In the variability analysis, there was also a significant module effect in intra-individual variability (*F*(6) = 50.12, *p* < 0.001; Figure 3A) and in inter-sub variability (*F*(6) = 364.70, *p* < 0.001; Figure 3B). The following Tukey HSD test revealed the module differences in both intra- and inter-sub variability. Results indicated that the limbic module showed the highest intra- and inter-sub variability, followed by the control, dorsal, and ventral attention modules, and then the DMN module, while the visual and somatomotor modules showed the least intra- and inter-sub variability.

### 3.3. Individual variation comparison between resting and language task state functional network

**Figure 4:**
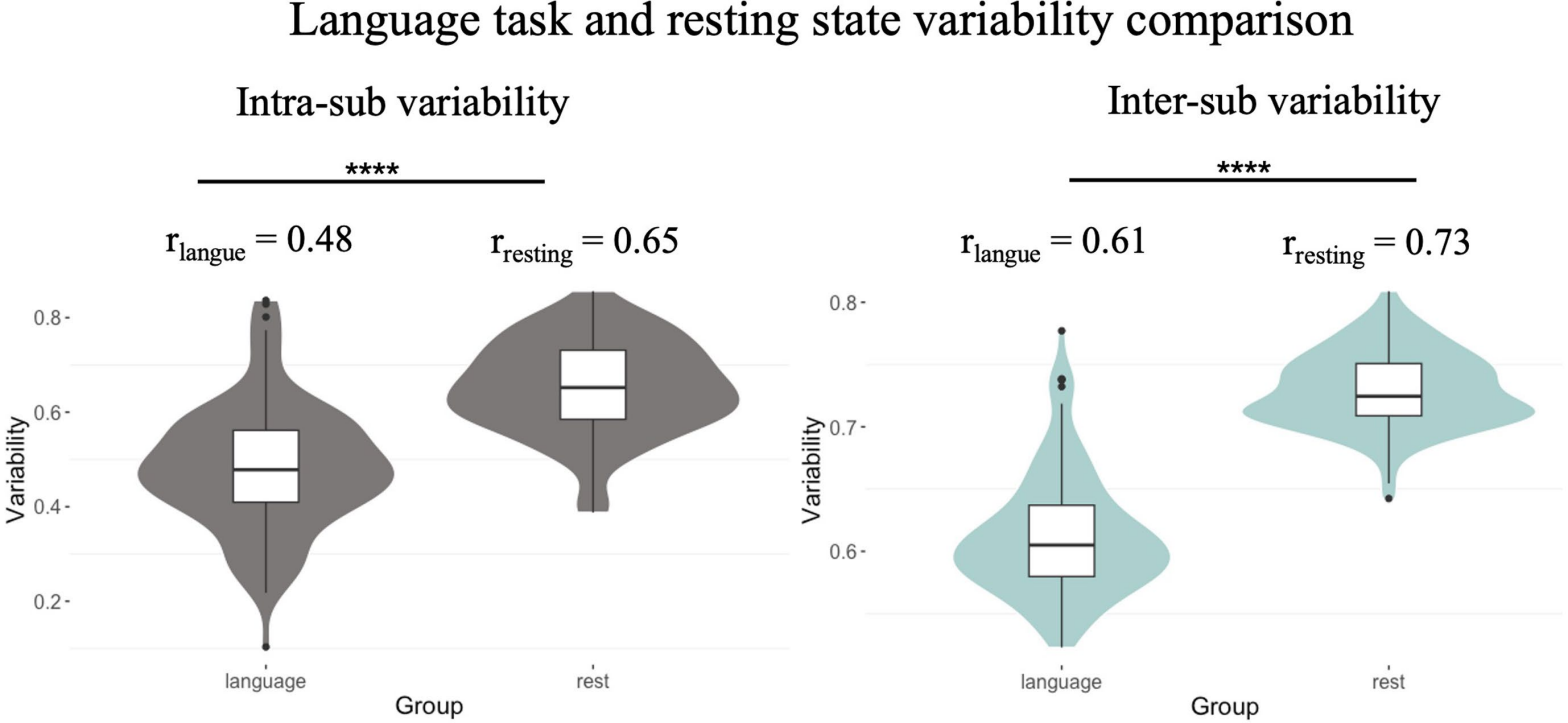
Individual variability comparison between resting and language task state. Intra-sub and inter-sub variability in the language task (*r* = 0.48, *r* = 0.61) was less than in the resting state (*r* = 0.65, *r* = 0.73, respectively).

We examined language task and resting state differences using a paired sample t-test. The results indicated that the intra-sub variability in the language task (mean *r* = 0.48) was less than in the resting state (mean *r* = 0.65; two-tailed paired Student’s *t*-test: T(99) = −9.18, *p* < 0.001; Figure 4, left). In addition, the inter-sub variability in the language task (mean *r* = 0.61) was less than in the resting state (mean *r* = 0.73; two-tailed paired Student’s *t*-test: T(99) = −19.11, *p* < 0.001; Figure 4, right). These results indicated that the language task can constrain both intra- and inter-individual variability in functional brain architecture.

We also compared the intra- and inter-sub variability ranking of all modules in task and resting state by carrying out a Kendall’s Concordance (Kendall’s w test; intra-sub variability: Kendall’s W(6) = 0.80, *p* = 0.141; inter-sub variability: Kendall’s W(6) = 0.77, *p* = 0.162), the result indicated that ranking consistency of both the intra- and inter-sub variability between task and resting state is strong, but not significant.

**Figure 5:**
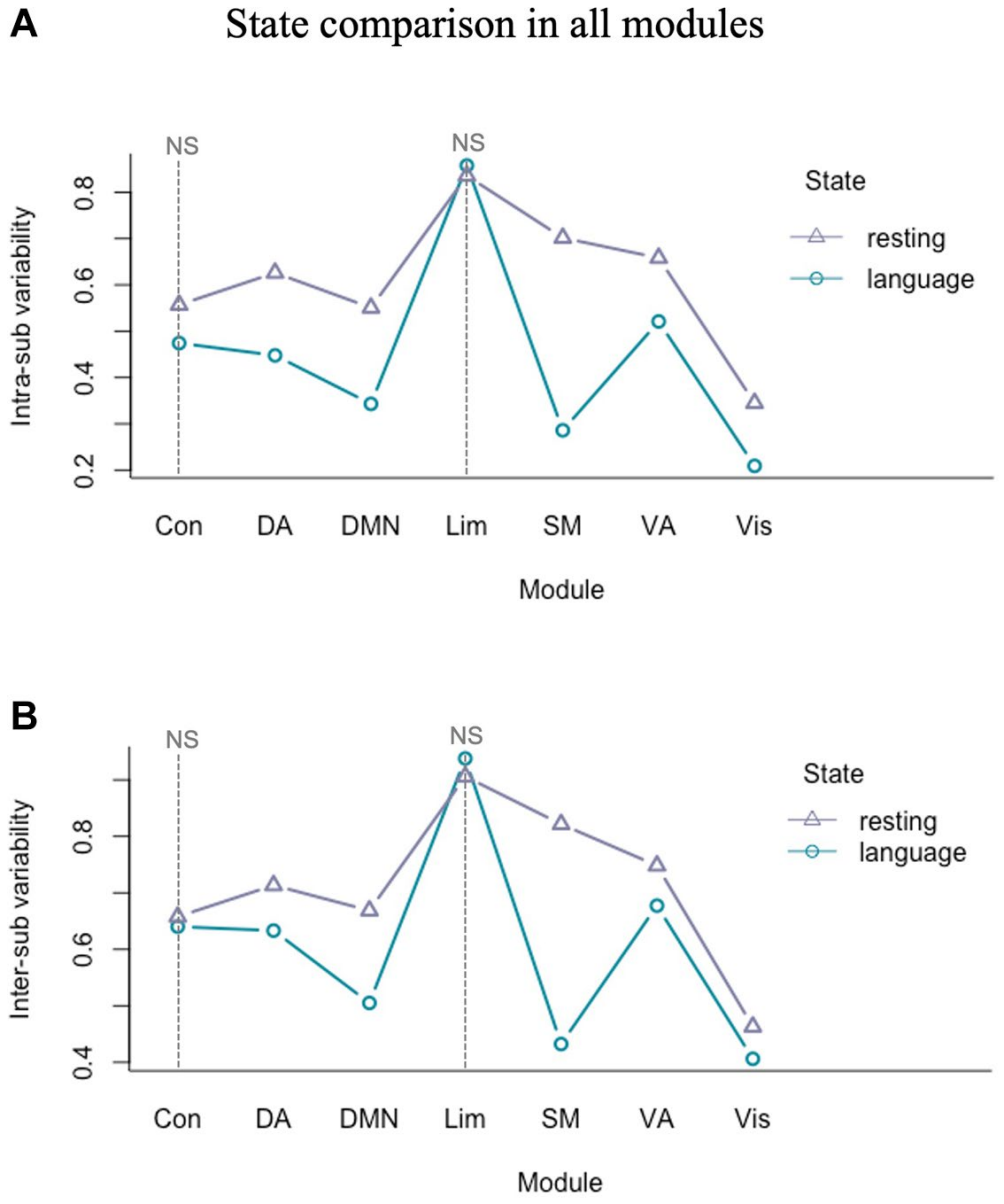
Intra- and inter-sub variability comparison of all modules in language task and resting state. **(A)** The intra-sub variability of all modules in the language task was significantly less than in the resting state except for the limbic and control modules. **(B)** The inter-sub variability of all modules in the language task was significantly less than in the resting state except for the limbic and control modules. NS, not significant.

We further carried out a 2 (resting/task) by 7 (visual/somatomotor/dorsal attention/ventral attention/limbic/control/DMN) two-factor ANOVA to investigate the state and module effect on intra- and inter-sub variability. The results indicated that there were significant main effects of state and module, and also a significant interaction effect of state by module in both intra-sub (state effect: *F*(1) = 123.27, *p* < 0.001; module effect: *F*(6) = 78.73, *p* < 0.001; state by module interaction effect: *F*(6) = 12.07, *p* < 0.001) and inter-sub variability (state effect: *F*(1) = 589.80, *p* < 0.001; module effect: *F*(6) = 636.50, *p* < 0.001; state by module interaction effect: *F*(6) = 141.60, *p* < 0.001). The intra- (Figure 5A) and inter-sub variability (Figure 5B) of all modules in the language task were significantly less than in the resting state except for the limbic and control modules, which were insignificant. Specifically, the DMN, somatomotor, and attention modules showed the most significant language versus resting difference.

## 4. Discussion

In this study, we explored the individual uniqueness of language task-evoked functional brain network and compared the network uniqueness between language task and resting state. The main findings are as follows: (1) The intrinsic and language task-induced functional networks exhibited remarkable differences across individuals; the story comprehension task can constrain both intra- and inter-individual variability in the functional brain network. (2) Intra- and inter-individual variability of language task and resting state functional networks were spatially heterogeneous, and the intra- and inter-individual differences ranking of language task and resting state is mainly consistent.

### 4.1. Resting and language task state functional networks are unique in individuals

In this study, we demonstrated that individual functional networks of two states are unique, and language task states can constrain individual functional brain network variability. Prior studies have indicated that the brain’s resting state functional networks are stable and personalized (Finn et al., 2015, 2017; Liao et al., 2017; Wang et al., 2021; Sbaihat et al., 2022). However, we know relatively little about the individual variability characteristics of the language task-evoked functional network. As an expansion of previous studies, we demonstrated that a language task-evoked functional network is also unique, as evidenced by remarkable inter-individual differences observed in it. Moreover, we observed that the individual variability of the functional network in the language task was less than in the resting state; this indicated that the language task state could constrain individual functional brain network variability. This is consistent with a previous study which demonstrated the existence of a task-general network architecture distinguishing task states from rest, and the brain’s functional network architecture during task performance is shaped by evoked task-general and task-specific network changes (Cole et al., 2014). Moreover, a study also indicated that functional connectivity variability was more significant during rest than during tasks, suggesting increased mind wandering at resting state (Elton and Gao, 2015). In addition, Studies comparing individual trait prediction from rest and task conditions also pointed out that task-evoked brain state manipulation can improve individual cognition prediction (Greene et al., 2018; Jiang et al., 2020). These findings implicated that task-induced changes in functional connectivity not only subserved the performance of the task at hand, but also offered a constrained manipulation of the brain state that taps into relevant circuitry (Greene et al., 2018). Conversely, the resting state is messy and unconstrained, and patterns of the functional network derived from it likely reflect many influences, which may introduce more individual variability.

### 4.2. Individual variability of language task and resting state functional networks are spatially heterogeneous

Both intra- and inter-individual variability of language task and resting state functional networks were spatially heterogeneous, with the limbic module manifesting the highest individual variability, followed by the control, dorsal, and ventral attention modules. In contrast, the visual modules showed the most minor individual variability. This rank agrees primarily with reported findings concerning patterns of the resting-state functional network, that is, more variability was mainly observed in the high-level association cortices, while lower variability was observed in the primary areas (Gordon et al., 2017; Liao et al., 2017; Wang et al., 2021). This rank is also consistent with a study estimating individual variability in regional activity under a vocal or non-vocal sound listening task (Ren et al., 2021), which proposed an argument that the inter-individual variability gradient reflected functional processing hierarchy; that is, the individual variability of regional activity was less in low-level auditory processing regions of Heschl’s gyrus and sulcus but much greater in high-level regions of superior temporal gyrus and planum temporale (Ren et al., 2021). The high inter-individual variability of functional networks in high-level association areas may be attributed to their relatively late maturation trajectory (Hill et al., 2010; Mueller et al., 2013), and hence, they are more likely to be affected by environmental factors which are characterized by more individual variability. Conversely, the function of unimodal sensory processing areas is primarily established at a very early age, changing only modestly thereafter; hence, it may be less influenced by the environment and characterized by less individual variability.

In addition, we demonstrated that modular rankings of individual differences are mainly consistent across the language task and resting states. In both states, the limbic module manifested the most significant individual difference, followed by the control and attention modules, while the visual modules showed the most minor individual variability. Only the sensorimotor module showed state unconformity, which showed minor individual variability in the language task state while relatively greater individual variability in the resting state. This result is inconsistent with previous findings, which indicated that sensorimotor systems were the least variable in resting state functional connectivity (Mueller et al., 2013). This inconsistency may be attributed to the fact that different ages of participants were recruited in these two studies. In the study by Mueller et al. (2013), functional network variability was estimated by taking a dataset with relative older age (mean age of 51.8). Their findings were also supported by a study investigating individual variability in functional connectivity in elderly individuals, which indicated that sensorimotor structures exhibited minimal variability among individuals (Li et al., 2017). Participants in our study were relatively younger (mean age of 28.5), which may induce this inconsistency in comparing the results. However, further research still needs to directly compare the young and old brains to reveal the modular hierarchy of inter-individual functional connectivity variability.

Moreover, we found that the individual variability of all modules in the language task was less than in the resting state, except for the limbic and control modules. As mentioned above, it is unsurprising that the task state can constrain individual functional brain network variability because the resting state is messy, whereas the task state is controlled. We gave further evidence that the limbic and control modules failed to show state differences in functional network variability comparison. According to previous findings, the control and limbic networks consistently displayed high inter-individual variability in the resting state functional network (Mueller et al., 2013; Xu et al., 2019). This may indicate a domain-general pattern of individual variability distribution.

### 4.3. Limitation

Several issues need to be considered in further studies. First, we observed that the sensorimotor module showed state unconformity in functional network variability, with the most minor individual variability in the language task state, and relatively greater individual variability in the resting state. This may be due to the age differences between this study and previous works. Further research needs to take the age effect into account to reveal a modular hierarchy of functional connectivity variability more comprehensively. Second, the task design in this study did not manipulate any psycholinguistic variables; hence, it might be less refined in interpreting individual variability in language comprehension. This limitation should be addressed in follow-up studies. Last, the data set we used in the current study came from the HCP 100 unrelated subjects, which ensures that all participants are not family relatives. This criterion was crucial in our study to exclude the need for family-structure co-variables in our analyses, preventing us from using the larger sample datasets. Future studies can be performed on larger study samples to validate these results.

## 5. Conclusion

In this study, we used T1 anatomical and functional brain imaging data from the Human Connectome Project (HCP) to characterize the uniqueness of the functional network in both task and resting states. We first demonstrated that the intrinsic and task-induced functional networks exhibited remarkable differences across individuals, but the language task can constrain inter-individual variability in functional brain networks. Furthermore, we found that the inter-individual variability of functional networks in two states was broadly consistent and spatially heterogeneous, with high-level association areas manifesting greater variability than primary sensory processing areas. Our results suggested that the functional network underlying language comprehension is unique at the individual level, and the inter-individual variability architecture of the functional network is mainly consistent in language task and resting state.

## Data availability statement

The original contributions presented in the study are included in the article/supplementary material, further inquiries can be directed to the corresponding author.

## Ethics statement

The studies involving humans came from a published dataset named Human Connectome Project: https://www.humanconnectome.org/study/hcp-young-adult/document/1200-subjects-data-release. The studies were conducted in accordance with the local legislation and institutional requirements. The participants provided their written informed consent to participate in this study.

## Author contributions

XL: Conceptualization, Formal analysis, Funding acquisition, Investigation, Methodology, Project administration, Visualization, Writing – original draft. LY: Conceptualization, Writing – review & editing.

## Footnotes

^1^ https://www.humanconnectome.org/study/hcp-young-adult/document/1200-subjects-data-release

^2^ https://www.humanconnectome.org/study/hcp-young-adult/document/1200-subjects-data-release
