# Supplementary figures and images for: Individual differences in the language task-evoked and resting-state functional networks
Source: Front Hum Neurosci. 2023 Nov 2;17:1283069. doi: 10.3389/fnhum.2023.1283069 (PMC10656779; doi:10.3389/fnhum.2023.1283069)

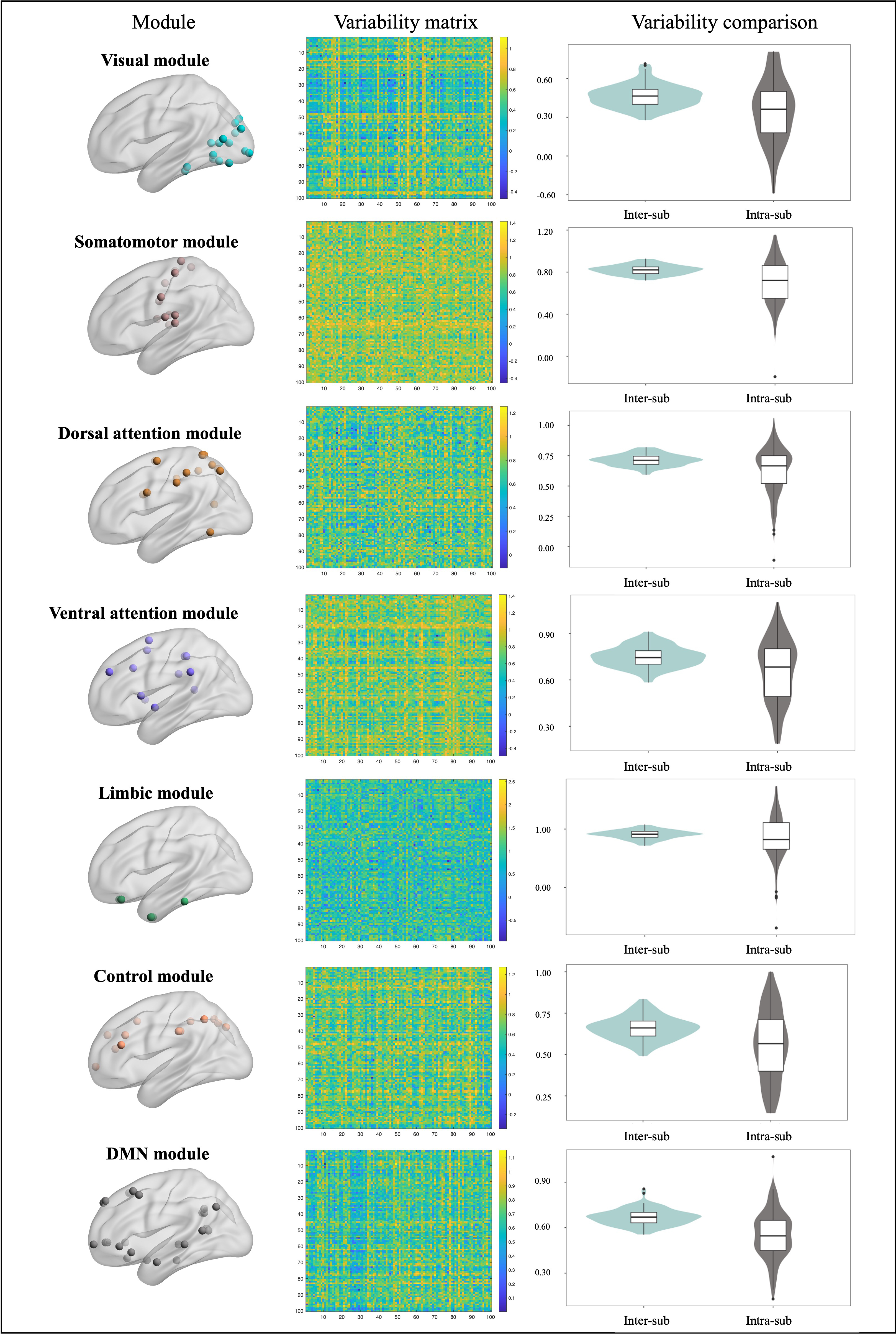

Supplement: Supplementary file 1 [file Image_1.jpg]

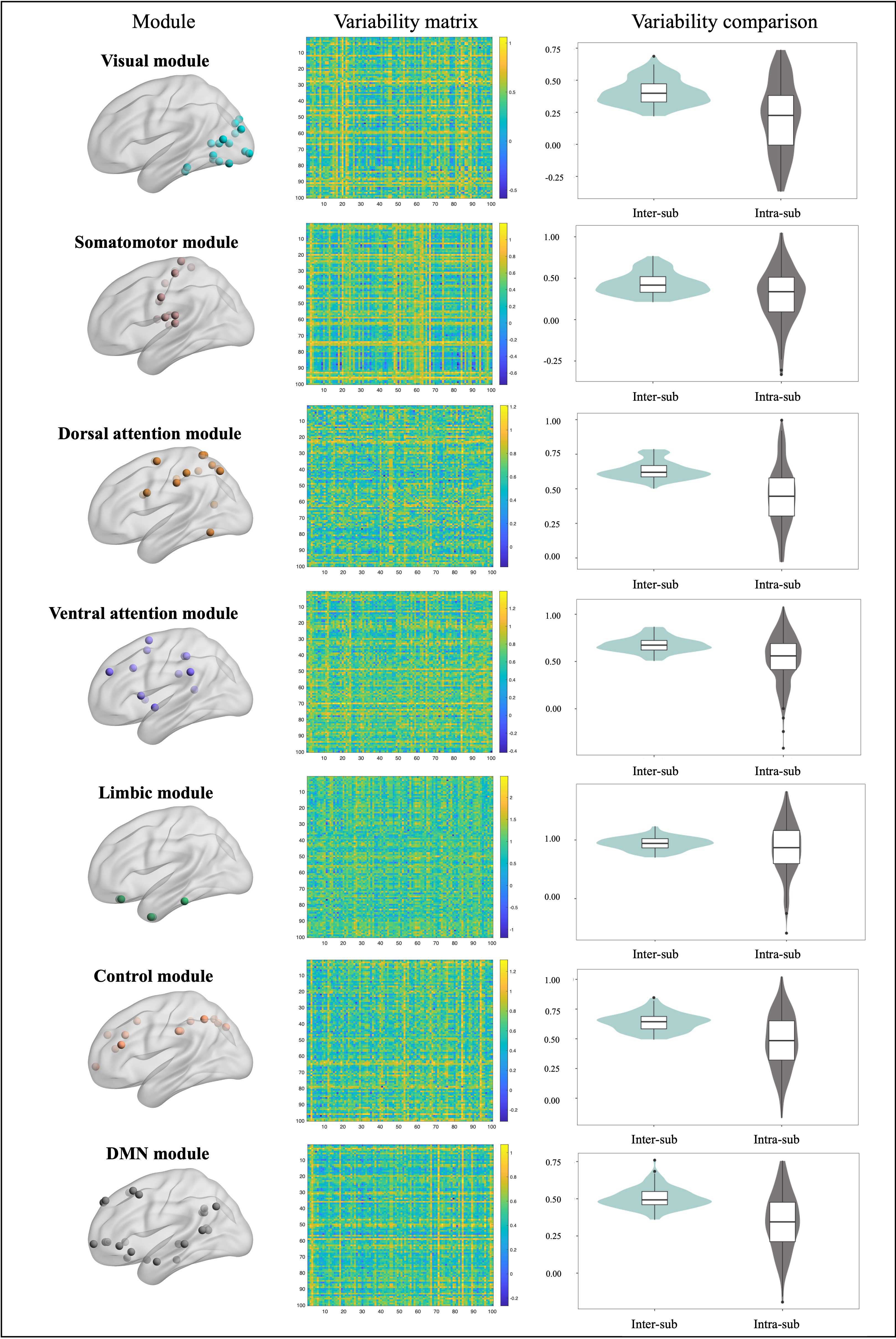

Supplement: Supplementary file 2 [file Image_2.jpg]
