# Supplementary material for: Individual differences in the language task-evoked and resting-state functional networks
Source: Front Hum Neurosci. 2023 Nov 2;17:1283069. doi: 10.3389/fnhum.2023.1283069 (PMC10656779; doi:10.3389/fnhum.2023.1283069)
Supplement: Supplementary file 3 [file Table_1.docx]

Supplementary table. Intra-sub and inter-sub variability comparison results in all modules in resting and language task states.

|  | Resting | | | | Language | | | |
| --- | --- | --- | --- | --- | --- | --- | --- | --- |
|  | inter-sub variability | intra-sub variability | T | *p* | inter-sub variability | intra-sub variability | T | *p* |
| Visual module | 0.46 | 0.34 | 6.15 | <0.001 | 0.41 | 0.21 | 8.86 | <0.001 |
| Somatomotor module | 0.82 | 0.7 | 5.99 | <0.001 | 0.43 | 0.29 | 5.93 | <0.001 |
| Dorsal attention module | 0.71 | 0.63 | 4.88 | <0.001 | 0.63 | 0.45 | 9.72 | <0.001 |
| Ventral attention module | 0.75 | 0.66 | 5.03 | <0.001 | 0.68 | 0.52 | 6.76 | <0.001 |
| Limbic module | 0.91 | 0.84 | 1.84 | 0.05 | 0.94 | 0.86 | 1.95 | 0.05 |
| Control module | 0.66 | 0.57 | 5.64 | <0.001 | 0.64 | 0.47 | 8.09 | <0.001 |
| DMN module | 0.67 | 0.55 | 8.6 | <0.001 | 0.51 | 0.34 | 10.51 | <0.001 |
